# (*S*)-2-[(4-Fluoro­phen­yl)formamido]-3-phenyl­propanoic acid

**DOI:** 10.1107/S2414314620008974

**Published:** 2020-07-10

**Authors:** Kathleen S. Lee, Luke Turner, Cynthia B. Powell, Eric W. Reinheimer

**Affiliations:** a Department of Chemistry and Biochemistry, Abilene Christian University, ACU 28132, Abilene, Texas 79699, USA; b Rigaku Americas Corporation, 9009 New Trails Drive, The Woodlands, Texas, 77381, USA; Vienna University of Technology, Austria

**Keywords:** crystal structure, amino acid derivative, solid phase synthesis, hydrogen bonding

## Abstract

The title compound, C_16_H_14_FNO_3_, was synthesized *via* solid phase methods; it exhibits monoclinic (*P*2_1_) symmetry at room temperature. The two independent mol­ecules that comprise the asymmetric unit display distinct torsion angles of 173.2 (2) and 72.6 (2)° along the central *sp* ^3^ C—N bond. In the crystal, hydrogen bonding through N—H⋯O contacts couples the asymmetric unit mol­ecules into pairs that align in layers extending parallel to (100) *via* additional O—H⋯O inter­actions. The phenyl ring of one independent mol­ecule was found to be disordered over two sets of sites in a 0.55 (3):0.45 (3) ratio.

## Abstract 2

The crystal structure exhibits monoclinic (*P*2_1_) symmetry at room temperature. The two mol­ecules in the asymmetric unit of the title compound, C_16_H_14_FNO_3_, exhibit different torsion angles along the central *sp* ^3^ C—N bonds and are linked together through two N—H⋯O hydrogen-bonding inter­actions.

## Graphical abstract

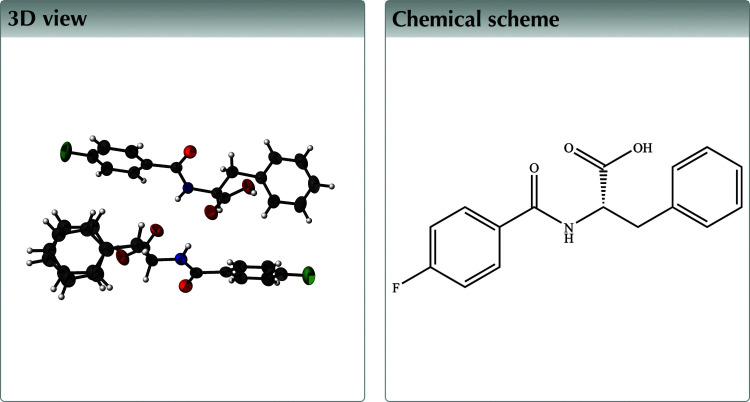

## Structure description

Anti­biotic resistance is a major global concern, compounded by the shortage of novel classes of anti­biotics in the clinical pipeline (Friedman *et al.*, 2016 ▸; Frieri *et al.*, 2017 ▸). In order to address the need for new pharmaceuticals and to incorporate drug discovery into the undergraduate curriculum, William Scott and coworkers created the Distributed Drug Discovery (D3) program (Scott & O’Donnell, 2009 ▸). D3′s virtual catalogs enumerate sets of amino-acid derivatives that have potential biological activity and that may be synthesized through straightforward combinatorial methods (Scott *et al.*, 2009 ▸; Abraham *et al.*, 2017 ▸). The D3 Lab 2 procedure targets *N*-acyl derivatives of natural amino acids in three steps (Dounay *et al.*, 2019 ▸). In this paper, we report the use of the D3 procedure to obtain the title compound as a single stereoisomer.

The compound was synthesized *via* solid phase methods starting from an Fmoc-protected phenyl­alanine bound to a Wang resin, which was purchased from CreoSalus Advanced Chem Tech as the enanti­opure *S* stereoisomer. The stereocenter remains unchanged during the deprotection, benzoyl­ation, cleavage sequence to form the final product. The absolute configuration of the title compound has been established by anomalous dispersion effects in the diffraction measurement.

**Figure 1:**
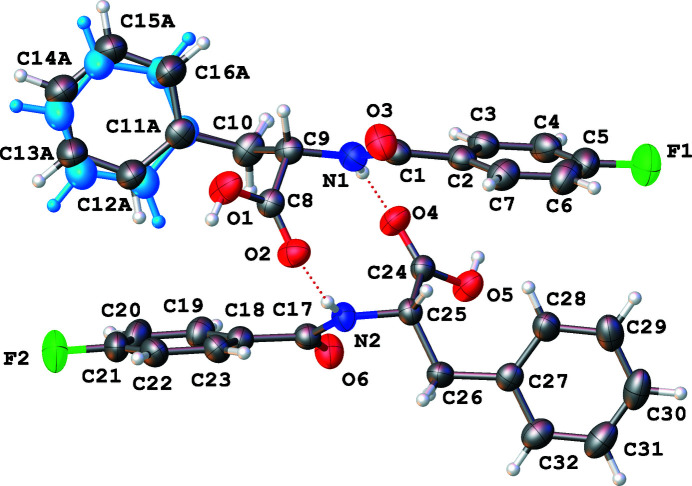
The asymmetric unit consists of two mol­ecules of the title compound, shown here with displacement ellipsoids drawn at the 50% probability level. The minor component of the disordered phenyl ring is shown in pale blue.

**Figure 2:**
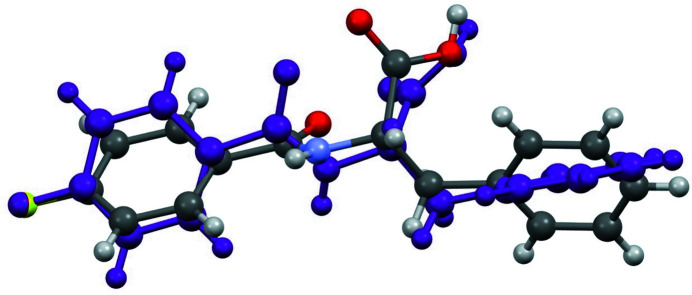
An overlay illustration of the two independent mol­ecules. For mol­ecule 1 only the major component of the disordered phenyl ring is displayed; mol­ecule 2 is shown in purple.

**Figure 3:**
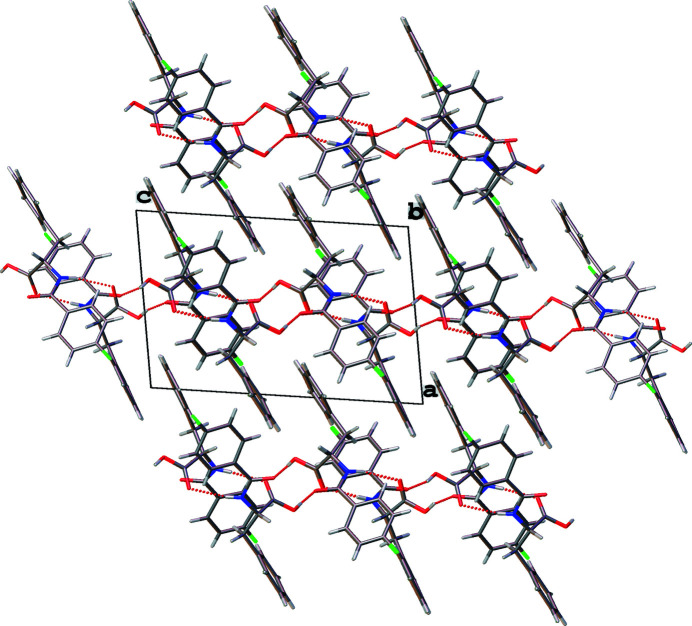
The packing of the mol­ecules of the title compound in a view along the *b* axis, showing hydrogen-bonded layers parallel to (100).

Two unique mol­ecules comprise the asymmetric unit. Within the mol­ecules the planes containing the benzene rings are rotated with respect to one another by 79.9 (1)° (mol­ecule 1, containing N1—F1) and 89.3 (1)° (mol­ecule 2, containing N2—F2), as shown in Fig. 1 ▸. The phenyl ring in mol­ecule 1 is disordered over two slightly different positions. Inter­estingly, torsion angles measured at the middle of the mol­ecules are quite different; in mol­ecule 1, the C1—N1—C9—C10 angle is 173.2 (2)° and in mol­ecule 2, the analogous C17—N2—C25—C26 angle is 72.6 (2)°. The amino-hydrogen atom on each mol­ecule is hydrogen-bonded to the organic-acid carbonyl O atom on the other mol­ecule (N—H⋯O contacts, Table 1 ▸) coupling the mol­ecules in the asymmetric unit so that they are positioned on top of each other with a fluoro-substituted ring above and nearly perpendicular to the unsubstituted ring of the partner mol­ecule [mean dihedral angle between the rings of the two mol­ecules = 87 (3)°]. Fig. 2 ▸ shows an overlay of the two distinct mol­ecules, highlighting their conformational differences. As shown in Fig. 3 ▸, the coupled asymmetric-unit pairs align in chains parallel to [010] *via* additional hydrogen-bonding inter­actions between OH organic-acid hydrogen atoms and the amide oxygen atoms in adjacent pairs through the O—H⋯O contacts listed in Table 1 ▸. Layers of chains extending parallel to (100) are visible when the packing is viewed along the *b* axis. The benzene-ring ends of the asymmetric-unit pairs that project out of each hydrogen-bonded chain occupy the voids between coupled asymmetric-unit pairs in the chain layers above and below, allowing closer packing.

## Synthesis and crystallization

50 µmol of *S*-phenyl­alanine protected with fluorenyl­methyl­oxycarbonyl (Fmoc) and bound to a Wang resin were placed in a fritted vial with screw caps at both ends. The resin was rinsed with three 3 ml aliquots of *N*-methyl-2-pyrrolidone (NMP) and three 2 ml aliquots of NMP:piperidine (4:1). The bottom of the vial was capped to prevent the acyl­ating reagents from draining from the vial. To the resin was added 1.0 ml of a solution of *p*-fluoro­benzoic acid (0.25 *M*) and hy­droxy­benzotriazole (HOBt, 0.25 *M*) in NMP and 0.5 ml of 0.5 *M* diiso­propyl­carbodi­imide in NMP. The vial was capped, inverted three times, and allowed to sit. After four days, the vial was uncapped at both ends, and the resin was washed sequentially with two 3 ml portions of NMP, two 3 ml portions of tetra­hydro­furan, and three 3 ml portions of di­chloro­methane. The reaction vial was placed over a collection vial, and the resin was washed twice with 2.0 ml of tri­fluoro­acetic acid–di­chloro­methane–water (35:60:5) and once with 2.0 ml of di­chloro­methane. The collection vial containing the combined rinses was placed in a vacuum chamber to remove the solvents. Column chromatography (hexa­nes:acetone, 75/25 *v*/*v*) afforded 12.8 mg of the title compound (89% overall yield from Fmoc–Phe–Wang resin). The purified product was crystallized from a di­chloro­methane solution layered with a solution of heptane and benzene. ^1^H NMR (600 MHz, CDCl_3_/CD_3_OD) δ 7.73 (*m*, 2H), 7.13 (*m*, 3H), 7.10 (*m*, 4H), 5.05 (*t*, *J* = 5.6 Hz, 1H), 3.27 (*dd*, *J* = 13.8, 5.7 Hz, 1H), 3.21 (*dd*, *J* = 13.8, 5.6 Hz, 1H). ^13^C NMR (150 MHz, CDCl_3_/CD_3_OD) δ 172.23, 166.11, 164.09, 135.77, 129.49, 129.43, 129.29, 128.67, 127.26, 115.78, 53.55, 37.78.

## Refinement

Crystal data, data collection and structure refinement details are summarized in Table 2 ▸. The phenyl ring (C11–C16) of mol­ecule 1 was found to be disordered over two sets of sites in a ratio of 0.55 (3):0.45 (3). The AFIX 66 constraint was applied to both parts of the disordered phenyl ring, and the RIGU rigid body restraint was applied to all non-hydrogen atoms in those rings with σ values of 0.001 for the 1–2 and 1–3 distances.

## Supplementary Material

Crystal structure: contains datablock(s) I. DOI: 10.1107/S2414314620008974/wm4133sup1.cif

Supporting information file. DOI: 10.1107/S2414314620008974/wm4133Isup2.cml

CCDC reference: 1975451

Additional supporting information:  crystallographic information; 3D view; checkCIF report

## Figures and Tables

**Table 1:** Hydrogen-bond geometry (Å, °)

*D*—H⋯*A*	*D*—H	H⋯*A*	*D*⋯*A*	*D*—H⋯*A*
O5—H5⋯O6^i^	0.84	1.83	2.6497 (18)	163
O1—H1⋯O3^ii^	0.84	1.77	2.607 (2)	177
N1—H1*A*⋯O4	0.89 (2)	2.16 (2)	3.039 (2)	170 (2)
N2—H2⋯O2	0.84 (2)	2.06 (2)	2.900 (2)	170 (2)

Symmetry codes: (i) 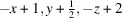 ; (ii) 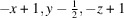 .

**Table 2:** Experimental details

Crystal data	
Chemical formula	C_16_H_14_FNO_3_
*M*_r_	287.28
Crystal system, space group	Monoclinic, *P*2_1_
Temperature (K)	100
*a*, *b*, *c* (Å)	9.74134 (5), 9.83313 (4), 14.91737 (6)
β (°)	98.2662 (4)
*V* (Å^3^)	1414.06 (1)
*Z*	4
Radiation type	Cu *K*α
μ (mm^−1^)	0.86
Crystal size (mm)	0.56 × 0.21 × 0.12

Data collection	
Diffractometer	Rigaku Oxford Diffraction SuperNova, Dual, Cu at home/near, AtlasS2
Absorption correction	Gaussian (*CrysAlis PRO*; Rigaku OD, 2019 ▸)
*T*_min_, *T*_max_	0.452, 1.000
No. of measured, independent and observed [*I* > 2σ(*I*)] reflections	53742, 5671, 5602
*R*_int_	0.032
(sin θ/λ)_max_ (Å^−1^)	0.622

Refinement	
*R*[*F* ^2^ > 2σ(*F* ^2^)], *wR*(*F* ^2^), *S*	0.028, 0.079, 1.05
No. of reflections	5671
No. of parameters	421
No. of restraints	355
H-atom treatment	H atoms treated by a mixture of independent and constrained refinement
Δρ_max_, Δρ_min_ (e Å^−3^)	0.16, −0.12
Absolute structure	Flack *x* determined using 2580 quotients [(*I* ^+^)−(*I* ^−^)]/[(*I* ^+^)+(*I* ^−^)] (Parsons *et al.*, 2013 ▸)
Absolute structure parameter	−0.05 (3)

Computer programs: *CrysAlis PRO* (Rigaku OD, 2019 ▸), *SHELXT* (Sheldrick, 2015*a* ▸), *SHELXL* (Sheldrick, 2015*b* ▸), *OLEX2* (Dolomanov *et al.*, 2009 ▸) and *publCIF* (Westrip, 2010 ▸).

## full crystallographic data

### Crystal data

**Table:**

C_16_H_14_FNO_3_	*F*(000) = 600
*M**_r_* = 287.28	*D*_x_ = 1.349 Mg m^−^^3^
Monoclinic, *P*2_1_	Cu *K*α radiation, λ = 1.54184 Å
*a* = 9.74134 (5) Å	Cell parameters from 40752 reflections
*b* = 9.83313 (4) Å	θ = 4.6–73.5°
*c* = 14.91737 (6) Å	µ = 0.86 mm^−^^1^
β = 98.2662 (4)°	*T* = 100 K
*V* = 1414.06 (1) Å^3^	Block, colourless
*Z* = 4	0.56 × 0.21 × 0.12 mm

### Data collection

**Table:**

Rigaku Oxford Diffraction SuperNova, Dual, Cu at home/near, AtlasS2 diffractometer	5671 independent reflections
Radiation source: micro-focus sealed X-ray tube, SuperNova (Cu) X-ray Source	5602 reflections with *I* > 2σ(*I*)
Mirror monochromator	*R*_int_ = 0.032
Detector resolution: 5.2387 pixels mm^-1^	θ_max_ = 73.7°, θ_min_ = 4.6°
ω scans	*h* = −11→12
Absorption correction: gaussian (*CrysAlis PRO*; Rigaku OD, 2019)	*k* = −12→12
*T*_min_ = 0.452, *T*_max_ = 1.000	*l* = −18→18
53742 measured reflections	

### Refinement

**Table:**

Refinement on *F*^2^	H atoms treated by a mixture of independent and constrained refinement
Least-squares matrix: full	*w* = 1/[σ^2^(*F*_o_^2^) + (0.0473*P*)^2^ + 0.1344*P*] where *P* = (*F*_o_^2^ + 2*F*_c_^2^)/3
*R*[*F*^2^ > 2σ(*F*^2^)] = 0.028	(Δ/σ)_max_ = 0.001
*wR*(*F*^2^) = 0.079	Δρ_max_ = 0.16 e Å^−^^3^
*S* = 1.05	Δρ_min_ = −0.12 e Å^−^^3^
5671 reflections	Extinction correction: SHELXL-2014/7 (Sheldrick 2014, Fc^*^=kFc[1+0.001xFc^2^λ^3^/sin(2θ)]^-1/4^
421 parameters	Extinction coefficient: 0.0068 (5)
355 restraints	Absolute structure: Flack *x* determined using 2580 quotients [(*I*^+^)-(*I*^-^)]/[(*I*^+^)+(*I*^-^)] (Parsons *et al.*, 2013)
Primary atom site location: dual	Absolute structure parameter: −0.05 (3)
Hydrogen site location: mixed	

### Special details

**Table:**

Geometry. All esds (except the esd in the dihedral angle between two l.s. planes) are estimated using the full covariance matrix. The cell esds are taken into account individually in the estimation of esds in distances, angles and torsion angles; correlations between esds in cell parameters are only used when they are defined by crystal symmetry. An approximate (isotropic) treatment of cell esds is used for estimating esds involving l.s. planes.

### Fractional atomic coordinates and isotropic or equivalent isotropic displacement parameters (Å^2^)

**Table:**

	*x*	*y*	*z*	*U*_iso_*/*U*_eq_	Occ. (<1)
O4	0.55896 (13)	0.60198 (15)	0.89050 (9)	0.0473 (3)	
O5	0.38969 (13)	0.57893 (16)	0.97497 (8)	0.0464 (3)	
H5	0.4369	0.6359	1.0082	0.070*	
O3	0.47124 (17)	0.78037 (16)	0.57577 (9)	0.0532 (4)	
O2	0.49339 (14)	0.45890 (15)	0.60468 (9)	0.0477 (3)	
N2	0.43835 (15)	0.38567 (14)	0.78447 (10)	0.0362 (3)	
O6	0.51088 (15)	0.27881 (15)	0.91538 (8)	0.0474 (3)	
O1	0.64625 (15)	0.49117 (17)	0.50928 (9)	0.0543 (4)	
H1	0.6083	0.4219	0.4837	0.081*	
F1	0.14855 (17)	1.06807 (19)	0.85687 (12)	0.0825 (5)	
F2	0.83586 (18)	−0.06630 (18)	0.65856 (14)	0.0876 (5)	
N1	0.59204 (17)	0.69008 (16)	0.69952 (10)	0.0401 (3)	
C18	0.59847 (18)	0.19811 (17)	0.78350 (12)	0.0387 (4)	
C25	0.35323 (17)	0.48177 (16)	0.82684 (10)	0.0334 (3)	
H25	0.3192	0.5505	0.7794	0.040*	
C8	0.59457 (18)	0.51500 (18)	0.58414 (11)	0.0370 (3)	
C24	0.44635 (16)	0.55878 (17)	0.90178 (10)	0.0334 (3)	
C17	0.51291 (18)	0.28963 (17)	0.83285 (11)	0.0360 (3)	
C23	0.5622 (2)	0.16621 (18)	0.69210 (14)	0.0439 (4)	
H23	0.4815	0.2053	0.6587	0.053*	
C1	0.4897 (2)	0.76851 (18)	0.65954 (11)	0.0399 (4)	
C27	0.11085 (18)	0.50384 (19)	0.87908 (11)	0.0380 (3)	
C2	0.3995 (2)	0.84212 (18)	0.71611 (12)	0.0408 (4)	
C26	0.22470 (19)	0.41301 (18)	0.85338 (13)	0.0421 (4)	
H26A	0.1839	0.3551	0.8021	0.050*	
H26B	0.2551	0.3519	0.9052	0.050*	
C22	0.6434 (2)	0.0778 (2)	0.64961 (16)	0.0532 (5)	
H22	0.6198	0.0564	0.5872	0.064*	
C32	−0.0009 (2)	0.4410 (2)	0.91043 (13)	0.0488 (4)	
H32	−0.0039	0.3446	0.9139	0.059*	
C9	0.67988 (19)	0.61896 (19)	0.64353 (11)	0.0383 (4)	
H9	0.7187	0.6866	0.6038	0.046*	
C19	0.7173 (2)	0.1409 (2)	0.83170 (15)	0.0495 (4)	
H19	0.7435	0.1631	0.8938	0.059*	
C10	0.8002 (2)	0.5494 (2)	0.70428 (12)	0.0483 (4)	
H10C	0.8406	0.6155	0.7507	0.058*	0.45 (3)
H10D	0.7619	0.4732	0.7363	0.058*	0.45 (3)
H10A	0.7621	0.4755	0.7385	0.058*	0.55 (3)
H10B	0.8443	0.6165	0.7488	0.058*	0.55 (3)
C21	0.7572 (2)	0.0226 (2)	0.69956 (18)	0.0567 (5)	
C28	0.1109 (2)	0.6446 (2)	0.87296 (14)	0.0457 (4)	
H28	0.1847	0.6897	0.8500	0.055*	
C31	−0.1082 (2)	0.5172 (3)	0.93667 (15)	0.0586 (5)	
H31	−0.1840	0.4727	0.9577	0.070*	
C4	0.3585 (2)	0.9521 (2)	0.85435 (15)	0.0539 (5)	
H4	0.3875	0.9752	0.9160	0.065*	
C7	0.2683 (2)	0.8821 (2)	0.67540 (14)	0.0514 (4)	
H7	0.2366	0.8567	0.6145	0.062*	
C29	0.0034 (2)	0.7198 (2)	0.90028 (15)	0.0553 (5)	
H29	0.0051	0.8163	0.8966	0.066*	
C3	0.4435 (2)	0.8773 (2)	0.80636 (13)	0.0461 (4)	
H3	0.5325	0.8497	0.8350	0.055*	
C16B	0.9064 (12)	0.3556 (8)	0.6455 (9)	0.063 (2)	0.45 (3)
H16B	0.8344	0.3050	0.6667	0.076*	0.45 (3)
C15B	1.0046 (15)	0.2896 (8)	0.6023 (8)	0.071 (2)	0.45 (3)
H15B	0.9998	0.1938	0.5939	0.085*	0.45 (3)
C14B	1.1099 (13)	0.3636 (14)	0.5712 (6)	0.064 (2)	0.45 (3)
H14B	1.1770	0.3184	0.5417	0.077*	0.45 (3)
C13B	1.1169 (8)	0.5037 (13)	0.5835 (7)	0.073 (2)	0.45 (3)
H13B	1.1889	0.5543	0.5623	0.087*	0.45 (3)
C12B	1.0187 (10)	0.5698 (8)	0.6268 (7)	0.054 (2)	0.45 (3)
H12B	1.0235	0.6655	0.6351	0.065*	0.45 (3)
C11B	0.9134 (10)	0.4957 (8)	0.6578 (9)	0.0461 (19)	0.45 (3)
C6	0.1834 (2)	0.9586 (3)	0.72273 (17)	0.0600 (5)	
H6	0.0943	0.9871	0.6948	0.072*	
C5	0.2313 (2)	0.9922 (2)	0.81097 (16)	0.0565 (5)	
C20	0.7975 (2)	0.0519 (3)	0.78943 (18)	0.0608 (5)	
H20	0.8785	0.0119	0.8219	0.073*	
C30	−0.1054 (2)	0.6563 (3)	0.93251 (15)	0.0580 (5)	
H30	−0.1780	0.7084	0.9517	0.070*	
C16A	1.0068 (9)	0.5848 (5)	0.6299 (7)	0.068 (3)	0.55 (3)
H16A	0.9943	0.6783	0.6424	0.082*	0.55 (3)
C15A	1.1190 (7)	0.5437 (10)	0.5885 (6)	0.0672 (17)	0.55 (3)
H15A	1.1832	0.6092	0.5728	0.081*	0.55 (3)
C14A	1.1373 (7)	0.4068 (12)	0.5702 (5)	0.0602 (16)	0.55 (3)
H14A	1.2140	0.3788	0.5419	0.072*	0.55 (3)
C13A	1.0434 (10)	0.3110 (7)	0.5931 (6)	0.0617 (17)	0.55 (3)
H13A	1.0559	0.2175	0.5806	0.074*	0.55 (3)
C12A	0.9312 (9)	0.3521 (6)	0.6345 (7)	0.0571 (16)	0.55 (3)
H12A	0.8670	0.2866	0.6502	0.068*	0.55 (3)
C11A	0.9129 (8)	0.4889 (7)	0.6528 (7)	0.0437 (15)	0.55 (3)
H1A	0.594 (2)	0.665 (2)	0.7566 (16)	0.047 (6)*	
H2	0.452 (2)	0.397 (2)	0.7303 (16)	0.044 (6)*	

### Atomic displacement parameters (Å^2^)

**Table:**

	*U*^11^	*U*^22^	*U*^33^	*U*^12^	*U*^13^	*U*^23^
O4	0.0360 (6)	0.0599 (8)	0.0469 (7)	−0.0066 (5)	0.0088 (5)	−0.0043 (6)
O5	0.0425 (6)	0.0618 (8)	0.0359 (6)	−0.0085 (6)	0.0089 (5)	−0.0125 (6)
O3	0.0718 (10)	0.0542 (8)	0.0339 (6)	0.0109 (7)	0.0087 (6)	0.0057 (6)
O2	0.0519 (7)	0.0538 (8)	0.0388 (6)	−0.0108 (6)	0.0115 (5)	−0.0034 (5)
N2	0.0439 (8)	0.0371 (7)	0.0288 (6)	0.0068 (6)	0.0089 (5)	0.0016 (5)
O6	0.0582 (8)	0.0501 (7)	0.0348 (6)	0.0137 (6)	0.0094 (5)	0.0083 (5)
O1	0.0544 (8)	0.0741 (10)	0.0366 (6)	−0.0145 (7)	0.0135 (6)	−0.0184 (6)
F1	0.0859 (10)	0.0780 (10)	0.0923 (10)	0.0130 (8)	0.0431 (8)	−0.0129 (9)
F2	0.0745 (10)	0.0735 (10)	0.1229 (13)	0.0176 (8)	0.0420 (9)	−0.0265 (9)
N1	0.0499 (8)	0.0421 (7)	0.0286 (6)	−0.0001 (6)	0.0073 (6)	−0.0007 (6)
C18	0.0401 (8)	0.0325 (7)	0.0448 (8)	0.0001 (6)	0.0108 (6)	0.0036 (6)
C25	0.0363 (7)	0.0331 (7)	0.0308 (7)	0.0035 (6)	0.0052 (6)	0.0006 (6)
C8	0.0404 (8)	0.0428 (8)	0.0277 (7)	0.0012 (6)	0.0043 (6)	0.0015 (6)
C24	0.0333 (7)	0.0344 (7)	0.0324 (7)	0.0032 (6)	0.0047 (6)	0.0013 (6)
C17	0.0383 (8)	0.0346 (7)	0.0356 (7)	0.0000 (6)	0.0066 (6)	0.0027 (6)
C23	0.0464 (9)	0.0369 (8)	0.0492 (9)	0.0001 (7)	0.0104 (7)	−0.0049 (7)
C1	0.0498 (9)	0.0356 (8)	0.0347 (7)	−0.0046 (7)	0.0076 (6)	0.0018 (6)
C27	0.0348 (8)	0.0444 (8)	0.0340 (8)	−0.0006 (6)	0.0021 (6)	−0.0025 (6)
C2	0.0494 (9)	0.0346 (8)	0.0397 (8)	−0.0052 (7)	0.0108 (6)	0.0021 (6)
C26	0.0385 (8)	0.0357 (8)	0.0528 (10)	−0.0026 (6)	0.0095 (7)	−0.0025 (7)
C22	0.0548 (10)	0.0434 (9)	0.0649 (11)	−0.0047 (8)	0.0206 (8)	−0.0140 (9)
C32	0.0439 (9)	0.0611 (11)	0.0419 (9)	−0.0029 (8)	0.0078 (7)	0.0062 (8)
C9	0.0429 (8)	0.0433 (8)	0.0290 (7)	−0.0032 (7)	0.0067 (6)	−0.0016 (6)
C19	0.0444 (9)	0.0516 (10)	0.0537 (10)	0.0078 (8)	0.0111 (8)	0.0080 (8)
C10	0.0443 (9)	0.0643 (11)	0.0348 (8)	0.0009 (8)	0.0012 (7)	−0.0016 (8)
C21	0.0515 (10)	0.0446 (10)	0.0796 (12)	0.0025 (8)	0.0283 (9)	−0.0072 (9)
C28	0.0397 (9)	0.0441 (9)	0.0533 (10)	0.0010 (7)	0.0064 (8)	−0.0053 (7)
C31	0.0433 (10)	0.0867 (13)	0.0474 (10)	0.0006 (9)	0.0124 (8)	−0.0001 (10)
C4	0.0642 (11)	0.0496 (10)	0.0509 (10)	−0.0098 (8)	0.0190 (8)	−0.0115 (8)
C7	0.0518 (10)	0.0580 (11)	0.0453 (9)	−0.0001 (8)	0.0104 (7)	0.0068 (8)
C29	0.0485 (10)	0.0570 (11)	0.0589 (12)	0.0110 (8)	0.0026 (9)	−0.0134 (9)
C3	0.0527 (10)	0.0421 (9)	0.0443 (9)	−0.0075 (8)	0.0093 (7)	−0.0043 (7)
C16B	0.066 (4)	0.057 (3)	0.071 (4)	0.003 (2)	0.024 (3)	−0.004 (2)
C15B	0.075 (5)	0.055 (3)	0.090 (4)	0.002 (2)	0.036 (4)	−0.002 (3)
C14B	0.072 (4)	0.049 (4)	0.078 (4)	0.004 (3)	0.033 (3)	−0.004 (3)
C13B	0.075 (3)	0.050 (4)	0.103 (5)	0.000 (3)	0.049 (4)	−0.005 (3)
C12B	0.052 (3)	0.053 (3)	0.058 (4)	0.001 (2)	0.014 (3)	−0.006 (2)
C11B	0.045 (3)	0.057 (3)	0.036 (4)	0.0017 (18)	0.002 (3)	−0.002 (2)
C6	0.0547 (11)	0.0649 (13)	0.0633 (11)	0.0081 (10)	0.0183 (9)	0.0095 (9)
C5	0.0616 (11)	0.0490 (10)	0.0643 (11)	−0.0014 (9)	0.0274 (9)	−0.0010 (9)
C20	0.0477 (10)	0.0570 (11)	0.0810 (13)	0.0138 (9)	0.0205 (9)	0.0074 (10)
C30	0.0442 (10)	0.0827 (13)	0.0471 (10)	0.0104 (9)	0.0070 (8)	−0.0144 (9)
C16A	0.063 (3)	0.057 (3)	0.092 (6)	−0.006 (2)	0.031 (4)	−0.006 (2)
C15A	0.063 (3)	0.056 (3)	0.088 (4)	−0.009 (2)	0.029 (3)	−0.007 (2)
C14A	0.056 (3)	0.057 (3)	0.071 (3)	−0.008 (2)	0.023 (2)	−0.002 (3)
C13A	0.059 (3)	0.048 (2)	0.083 (4)	−0.0027 (19)	0.029 (2)	0.000 (2)
C12A	0.054 (3)	0.049 (2)	0.072 (4)	−0.0034 (16)	0.023 (3)	−0.0009 (18)
C11A	0.041 (2)	0.051 (2)	0.037 (3)	−0.0016 (15)	−0.001 (2)	−0.0007 (18)

### Geometric parameters (Å, º)

**Table:**

O4—C24	1.210 (2)	C10—H10A	0.9900
O5—H5	0.8400	C10—H10B	0.9900
O5—C24	1.307 (2)	C10—C11B	1.482 (6)
O3—C1	1.242 (2)	C10—C11A	1.546 (5)
O2—C8	1.207 (2)	C21—C20	1.372 (4)
N2—C25	1.460 (2)	C28—H28	0.9500
N2—C17	1.338 (2)	C28—C29	1.391 (3)
N2—H2	0.84 (2)	C31—H31	0.9500
O6—C17	1.239 (2)	C31—C30	1.369 (4)
O1—H1	0.8400	C4—H4	0.9500
O1—C8	1.310 (2)	C4—C3	1.382 (3)
F1—C5	1.354 (3)	C4—C5	1.371 (3)
F2—C21	1.364 (2)	C7—H7	0.9500
N1—C1	1.332 (2)	C7—C6	1.384 (3)
N1—C9	1.457 (2)	C29—H29	0.9500
N1—H1A	0.89 (2)	C29—C30	1.375 (3)
C18—C17	1.491 (2)	C3—H3	0.9500
C18—C23	1.394 (3)	C16B—H16B	0.9500
C18—C19	1.390 (3)	C16B—C15B	1.3900
C25—H25	1.0000	C16B—C11B	1.3900
C25—C24	1.534 (2)	C15B—H15B	0.9500
C25—C26	1.525 (2)	C15B—C14B	1.3900
C8—C9	1.520 (2)	C14B—H14B	0.9500
C23—H23	0.9500	C14B—C13B	1.3900
C23—C22	1.388 (3)	C13B—H13B	0.9500
C1—C2	1.490 (3)	C13B—C12B	1.3900
C27—C26	1.516 (3)	C12B—H12B	0.9500
C27—C32	1.390 (3)	C12B—C11B	1.3900
C27—C28	1.387 (3)	C6—H6	0.9500
C2—C7	1.391 (3)	C6—C5	1.372 (3)
C2—C3	1.396 (3)	C20—H20	0.9500
C26—H26A	0.9900	C30—H30	0.9500
C26—H26B	0.9900	C16A—H16A	0.9500
C22—H22	0.9500	C16A—C15A	1.3900
C22—C21	1.357 (3)	C16A—C11A	1.3900
C32—H32	0.9500	C15A—H15A	0.9500
C32—C31	1.388 (3)	C15A—C14A	1.3900
C9—H9	1.0000	C14A—H14A	0.9500
C9—C10	1.536 (3)	C14A—C13A	1.3900
C19—H19	0.9500	C13A—H13A	0.9500
C19—C20	1.384 (3)	C13A—C12A	1.3900
C10—H10C	0.9900	C12A—H12A	0.9500
C10—H10D	0.9900	C12A—C11A	1.3900
			
C24—O5—H5	109.5	C11A—C10—H10B	108.6
C25—N2—H2	120.2 (16)	F2—C21—C20	118.2 (2)
C17—N2—C25	121.44 (14)	C22—C21—F2	118.5 (2)
C17—N2—H2	117.7 (16)	C22—C21—C20	123.3 (2)
C8—O1—H1	109.5	C27—C28—H28	119.9
C1—N1—C9	118.99 (14)	C27—C28—C29	120.3 (2)
C1—N1—H1A	120.1 (15)	C29—C28—H28	119.9
C9—N1—H1A	119.1 (15)	C32—C31—H31	119.8
C23—C18—C17	122.54 (16)	C30—C31—C32	120.4 (2)
C19—C18—C17	118.14 (17)	C30—C31—H31	119.8
C19—C18—C23	119.32 (17)	C3—C4—H4	120.8
N2—C25—H25	106.4	C5—C4—H4	120.8
N2—C25—C24	108.77 (13)	C5—C4—C3	118.5 (2)
N2—C25—C26	111.36 (14)	C2—C7—H7	119.7
C24—C25—H25	106.4	C6—C7—C2	120.7 (2)
C26—C25—H25	106.4	C6—C7—H7	119.7
C26—C25—C24	116.82 (14)	C28—C29—H29	119.6
O2—C8—O1	124.23 (16)	C30—C29—C28	120.8 (2)
O2—C8—C9	123.89 (15)	C30—C29—H29	119.6
O1—C8—C9	111.86 (15)	C2—C3—H3	119.7
O4—C24—O5	124.25 (16)	C4—C3—C2	120.5 (2)
O4—C24—C25	121.64 (15)	C4—C3—H3	119.7
O5—C24—C25	114.01 (14)	C15B—C16B—H16B	120.0
N2—C17—C18	117.28 (14)	C15B—C16B—C11B	120.0
O6—C17—N2	120.79 (16)	C11B—C16B—H16B	120.0
O6—C17—C18	121.93 (15)	C16B—C15B—H15B	120.0
C18—C23—H23	119.8	C14B—C15B—C16B	120.0
C22—C23—C18	120.45 (19)	C14B—C15B—H15B	120.0
C22—C23—H23	119.8	C15B—C14B—H14B	120.0
O3—C1—N1	119.71 (17)	C15B—C14B—C13B	120.0
O3—C1—C2	120.83 (17)	C13B—C14B—H14B	120.0
N1—C1—C2	119.46 (15)	C14B—C13B—H13B	120.0
C32—C27—C26	117.39 (17)	C12B—C13B—C14B	120.0
C28—C27—C26	124.34 (17)	C12B—C13B—H13B	120.0
C28—C27—C32	118.28 (19)	C13B—C12B—H12B	120.0
C7—C2—C1	118.01 (17)	C13B—C12B—C11B	120.0
C7—C2—C3	119.05 (19)	C11B—C12B—H12B	120.0
C3—C2—C1	122.88 (18)	C16B—C11B—C10	112.9 (6)
C25—C26—H26A	107.9	C12B—C11B—C10	127.1 (6)
C25—C26—H26B	107.9	C12B—C11B—C16B	120.0
C27—C26—C25	117.55 (15)	C7—C6—H6	120.8
C27—C26—H26A	107.9	C5—C6—C7	118.4 (2)
C27—C26—H26B	107.9	C5—C6—H6	120.8
H26A—C26—H26B	107.2	F1—C5—C4	118.9 (2)
C23—C22—H22	120.9	F1—C5—C6	118.3 (2)
C21—C22—C23	118.3 (2)	C4—C5—C6	122.9 (2)
C21—C22—H22	120.9	C19—C20—H20	120.8
C27—C32—H32	119.6	C21—C20—C19	118.5 (2)
C31—C32—C27	120.8 (2)	C21—C20—H20	120.8
C31—C32—H32	119.6	C31—C30—C29	119.4 (2)
N1—C9—C8	109.88 (14)	C31—C30—H30	120.3
N1—C9—H9	108.9	C29—C30—H30	120.3
N1—C9—C10	109.69 (14)	C15A—C16A—H16A	120.0
C8—C9—H9	108.9	C15A—C16A—C11A	120.0
C8—C9—C10	110.57 (16)	C11A—C16A—H16A	120.0
C10—C9—H9	108.9	C16A—C15A—H15A	120.0
C18—C19—H19	119.9	C14A—C15A—C16A	120.0
C20—C19—C18	120.2 (2)	C14A—C15A—H15A	120.0
C20—C19—H19	119.9	C15A—C14A—H14A	120.0
C9—C10—H10C	108.3	C15A—C14A—C13A	120.0
C9—C10—H10D	108.3	C13A—C14A—H14A	120.0
C9—C10—H10A	108.6	C14A—C13A—H13A	120.0
C9—C10—H10B	108.6	C12A—C13A—C14A	120.0
C9—C10—C11A	114.5 (5)	C12A—C13A—H13A	120.0
H10C—C10—H10D	107.4	C13A—C12A—H12A	120.0
H10A—C10—H10B	107.6	C13A—C12A—C11A	120.0
C11B—C10—C9	116.0 (6)	C11A—C12A—H12A	120.0
C11B—C10—H10C	108.3	C16A—C11A—C10	113.7 (5)
C11B—C10—H10D	108.3	C12A—C11A—C10	126.2 (5)
C11A—C10—H10A	108.6	C12A—C11A—C16A	120.0

### Hydrogen-bond geometry (Å, º)

**Table:**

*D*—H···*A*	*D*—H	H···*A*	*D*···*A*	*D*—H···*A*
O5—H5···O6^i^	0.84	1.83	2.6497 (18)	163
O1—H1···O3^ii^	0.84	1.77	2.607 (2)	177
N1—H1*A*···O4	0.89 (2)	2.16 (2)	3.039 (2)	170 (2)
N2—H2···O2	0.84 (2)	2.06 (2)	2.900 (2)	170 (2)

Symmetry codes: (i) −*x*+1, *y*+1/2, −*z*+2; (ii) −*x*+1, *y*−1/2, −*z*+1.
